# In Vitro Production of Plant Secondary Metabolites: Validated Platforms and Emerging Opportunities in Tropical and Subtropical Plant Species

**DOI:** 10.3390/plants15213244

**Published:** 2026-10-22

**Authors:** Daniela Ortega-Ante, Jacqueline Alejandra Noboa-Velástegui, Daniel Alejandro Luna-Velasco, Mónica Jadan-Guerrero

**Affiliations:** 1 Departamento de Ciencias Exactas, Universidad de las Fuerzas Armadas ESPE, Sangolquí 171103, Ecuador; 2 Faculty of Experimental Sciences, Department of Animal, Plant and Ecological Biology, Universidad de Jaen, Campus de las Lagunillas, S/N, 23071 Jaen, Spain; 3 Independent Researcher, 48003 Bilbao, Spain; jacquenob@gmail.com; 4 PANIAM Research Unit, Engineering Department, Universidad Nacional de Chimborazo, Av. Antonio José de Sucre, Riobamba 060150, Ecuador; daniel.luna@unach.edu.ec; 5 Departamento de Ciencias de la Vida y de la Agricultura, Grupo de Biomedicina y Medicina Traslacional, BMT, Universidad de las Fuerzas Armadas ESPE, Sangolquí 171103, Ecuador; mbjadan@espe.edu.ec

**Keywords:** plant secondary metabolite, plant tissue culture, platform validation, tropical and subtropical biodiversity

## Abstract

Plant in vitro culture has emerged as a biotechnological approach for the sustainable production of high-value secondary metabolites. Despite advances in plant tissue culture and phytochemical research, converting biologically productive in vitro systems into reliable production platforms remains a major challenge because metabolite production alone does not necessarily reflect technological maturity. This review evaluates plant in vitro production platforms for secondary metabolites in tropical and subtropical plant species by distinguishing validated, partially validated, and emerging platforms according to their level of technological development. To support this assessment, we propose a technological validation framework based on complementary experimental evidence that distinguishes species-level scientific knowledge from platform-level technological validation. The analysis reveals substantial differences in platform development and identifies production reliability as the principal limitation preventing many systems from reaching more advanced stages of technological maturity. These findings indicate that technological maturity should be understood as a progressive process driven by the accumulation of complementary experimental evidence rather than isolated experimental achievements. Future advances in plant in vitro production will depend on integrating mechanistic understanding of secondary metabolite biosynthesis with process engineering approaches to develop robust, reproducible, and scalable production platforms, thereby facilitating the sustainable biotechnological exploitation of tropical and subtropical plant biodiversity.

## 1. Introduction

Plant secondary metabolites constitute a structurally and functionally heterogeneous group of specialized compounds involved in plant defense, adaptation, and survival to biotic and abiotic stress [1,2]. Beyond their ecological roles, these compounds represent a major source of high-value bioactive substances widely exploited in pharmaceutical, nutraceutical, and agro-industrial applications [3,4].

The tropical and subtropical regions harbor high biodiversity, serving as an important reservoir of secondary metabolites with significant biotechnological potential. Currently, the exploitation of these species is predominantly informed by traditional ethnobotanical knowledge, which facilitates the identification of bioactive molecules in plants growing under natural, open-field conditions [5,6]. However, this biological wealth faces significant challenges due to its dependence on environmental conditions, leading to inconsistent yields and variable phytochemical profiles [2,7,8]. In this context, the transition toward controlled in vitro production systems emerges as a relevant strategy, not only to optimize metabolite production but also to reduce pressure on natural ecosystems and support biodiversity conservation.

Plant in vitro culture platforms are biotechnological tools for optimizing the production of secondary metabolites [1], including callus and cell suspension cultures, clonal micropropagation, and organ-specific cultures, enabling the manipulation of growth conditions and metabolic activity under reproducible environments [4,9,10]. Their performance, however, is not consistent across systems because it depends on interactions among biosynthetic regulation, cellular organization, and the structural characteristics of each culture approach [11].

Different classes of metabolites require specific cellular contexts to sustain active biosynthetic pathways [12]. For instance, root-derived metabolites such as alkaloids are more efficiently produced in hairy root cultures, whereas phenolic compounds tend to respond more strongly to elicitation in undifferentiated platforms such as callus or suspension cultures [7,9,10].

Tropical and subtropical regions of Africa, Latin America, and Southeast Asia harbor substantial plant diversity, including medicinal species with recognized phytochemical and biotechnological potential [5,6]. This potential provides a relevant basis for examining the extent to which culture systems developed for species from these regions have progressed from in vitro culture establishment and initial metabolite characterization toward reliable production platforms. Accordingly, this review evaluates the technological development of in vitro production platforms for secondary metabolites in tropical and subtropical plant species. By distinguishing validated, partially validated, and emerging platforms according to complementary experimental evidence, the review identifies their current stages of development, remaining technological gaps, and priorities for future research.

## 2. Methods

This review was conducted as a narrative bibliographic review. A literature search was conducted in PubMed and Scopus covering publications from 2000 to April 2026 (search conducted between 15 January and 17 April 2026). The search strings were adapted to each database’s syntax because the inclusion of the term “species” in the geographic descriptor produced zero results in PubMed; accordingly, broader geographic terms were used in that database while more specific terms were retained in Scopus.

PubMed: (“in vitro plant culture” OR “plant tissue culture” OR “plant cell culture” OR “micropropagation”) AND (“secondary metabolites” OR “bioactive compounds” OR “phytochemicals” OR “specialized metabolites” OR “antioxidants” OR “alkaloids” OR “flavonoids” OR “terpenoids” OR “secondary metabolites production”) AND (“subtropical” OR “tropical”).

Scopus: (“in vitro plant culture” OR “plant tissue culture” OR “plant cell culture” OR “micropropagation”) AND (“secondary metabolites” OR “bioactive compounds” OR “phytochemicals” OR “specialized metabolites” OR “antioxidants” OR “alkaloids” OR “flavonoids” OR “terpenoids” OR “secondary metabolites production”) AND (“subtropical species” OR “tropical species”).

The search identified 87 records. Inclusion criteria comprised peer-reviewed articles published in English or Spanish that directly addressed in vitro culture systems for secondary metabolite production in plant species, including both specialized review articles and primary research articles reporting quantitative metabolite production data. Studies were excluded if they did not include an in vitro culture component, lacked metabolite-level outcomes, or were focused exclusively on agronomic or micropropagation endpoints without phytochemical analysis. The search and initial screening were conducted independently by two authors using the agreed search strings; consistency was verified by comparing the number of records retrieved in each database, which were identical across both searches, and final inclusion decisions were made jointly based on the eligibility criteria described above. Following screening, 43 sources were retained and 44 were excluded.

The retained sources were critically analyzed to identify the technological attributes most consistently reported in the literature as indicators of in vitro production platform development. Based on this analysis, a technological validation framework was established comprising four core attributes: culture establishment, quantitative metabolite determination, optimization of culture conditions, and evidence of production reliability. This framework was used to classify each species or culture system into one of three categories, validated, partially validated, or emerging, according to the level of experimental evidence available in the reviewed literature. The operational definitions of each category and the full description of the validation framework are provided in the following section.

## 3. Results and Discussion

The application of the literature search and selection criteria described in this section identified 24 tropical and subtropical plant species with documented in vitro secondary metabolite production systems, which were retained for technological assessment using the validation framework proposed in this review (Table 1). The identified species span 20 botanical families, with Apocynaceae, Acanthaceae, and Rubiaceae among the most represented. In terms of native distribution, the dataset includes species native to tropical regions (e.g., *Andrographis paniculata*, *Carica papaya*, *Mitragyna speciosa*), subtropical regions (e.g., *Gardenia jasminoides*, *Simmondsia chinensis*, *Zanthoxylum armatum*), and species with broad tropical–subtropical distribution (e.g., *Camellia sinensis*, *Gloriosa superba*, *Withania somnifera*). The target metabolites reported across the identified systems encompass all major classes of plant secondary metabolites, including alkaloids (*Catharanthus roseus*, *Mitragyna speciosa*), terpenoids (*Azadirachta indica*, *Gloriosa superba*), phenolic compounds (*Camellia sinensis*, *Rubus fraxinifolius*), and other specialized metabolites of pharmacological and industrial interest. Applying the validation framework to these 24 species revealed that five platforms were classified as validated, sixteen as partially validated, and three as emerging, indicating that most in vitro systems for tropical and subtropical species have reached intermediate stages of technological development but have not yet demonstrated the full complement of attributes required for production-oriented platform validation.

**Literature review and summary interpretation**

**Biosynthesis and Subcellular Organization of Major Plant Secondary Metabolites**

The biosynthesis of plant secondary metabolites involves coordinated enzyme-catalyzed reactions distributed across multiple subcellular compartments rather than confined to a single cellular location. This spatial organization reflects the biochemical requirements of individual enzymes, including substrate availability, cofactors, redox balance, and energy supply. Biosynthetic intermediates are frequently transported between organelles before undergoing further modification, accumulation, or storage, thereby linking metabolic pathways across several intracellular compartments [12].

Terpenoids constitute the largest class of plant secondary metabolites and are synthesized through the mevalonate (MVA) and methylerythritol phosphate (MEP) pathways [8]. The MVA pathway is primarily localized in the cytosol, whereas the MEP pathway operates in plastids, with metabolic exchange of intermediates between the two pathways. Subsequent modification reactions are largely associated with the endoplasmic reticulum, and many terpenoids are ultimately accumulated in specialized secretory structures or intracellular storage compartments [13,14]. Phenolic compounds are synthesized mainly through the shikimate–phenylpropanoid pathway [15,16,17]. The shikimate pathway is primarily localized in plastids, whereas the downstream phenylpropanoid pathway is organized between cytosolic enzymes and enzymes associated with the cytoplasmic surface of the endoplasmic reticulum. This spatial organization supports successive enzymatic reactions leading to the biosynthesis of diverse phenolic subclasses, including flavonoids, stilbenes, lignins, and anthocyanins [17,18,19].

Finally, alkaloids are a structurally diverse group of nitrogen-containing secondary metabolites primarily derived from amino acid precursors [4,20]. Their biosynthesis is likewise highly compartmentalized, with successive enzymatic reactions distributed across multiple subcellular compartments. Early biosynthetic steps occur in the cytosol and plastids, whereas key oxidative reactions are localized in peroxisomes. Subsequent modifications involve the endoplasmic reticulum and vacuoles, requiring coordinated intracellular trafficking of metabolic intermediates. In addition, several alkaloid pathways exhibit tissue-specific compartmentalization, in which intermediates or final products are transported between different organs or specialized cell types. These processes are coordinated by membrane transporters, which regulate metabolite translocation, sequestration and detoxification [12,21,22,23].

Collectively, these differences illustrate that secondary metabolite production in plant in vitro systems is governed by the interaction between biosynthetic pathway regulation, tissue specificity, and culture architecture. While some metabolite classes can be efficiently induced in undifferentiated systems, others require the preservation of tissue-specific organization to sustain pathway activity. Therefore, the selection of an appropriate in vitro platform must be guided by the biological requirements of the target metabolite, providing a conceptual basis for the comparative analysis of culture systems presented in the following sections [4].

**Plant In vitro Culture Platforms for Secondary Metabolite Production**

The technological development of plant in vitro production platforms is a progressive process that extends beyond the successful establishment of a culture system [24]. Although the detection of secondary metabolites confirms the biosynthetic competence of cultured tissues, it does not necessarily indicate that a platform has reached the level of maturity required for reliable metabolite production.

Establishing an in vitro plant culture system provides the biological foundation for developing metabolite production strategies. The generation of stable callus cultures, cell suspensions, adventitious roots, hairy roots, or organized tissues demonstrates that plant cells can be maintained under controlled conditions, providing experimental systems for evaluating and improving secondary metabolite production [11,25]. However, the establishment of the culture alone does not demonstrate that the system functions as a metabolite production platform. Instead, it provides an experimental basis for quantitative metabolite determination, comparison among different culture systems, and the implementation of strategies to improve metabolite accumulation and production efficiency [11,24].

Quantitative determination of the target metabolite is a critical stage in in vitro plant production platforms, as it provides objective evidence of a culture system’s productivity. While qualitative detection confirms the presence of a target metabolite, quantitative determination transforms metabolite production into an experimentally measurable parameter that allows the productive performance of an in vitro culture system to be objectively evaluated, monitored, and compared among culture systems, culture conditions, and independent studies [11,24,26]. Such measurements also provide the experimental basis for evaluating changes in metabolite productivity following culture optimization, elicitation, precursor feeding, selection of highly productive lines, and other production-enhancing strategies [4]. Metabolite concentration, productivity, and yield are reported as the principal parameters used to evaluate the performance of plant in vitro production systems, whereas qualitative evidence primarily demonstrates the biosynthetic potential of the culture system [6,24].

Metabolite production in plant in vitro culture systems rarely reaches its maximum biosynthetic potential under a single set of culture conditions, making experimental optimization a central component in the technological development of secondary metabolite production platforms. This stage is described as a systematic process for increasing metabolite concentration, productivity, and yield by evaluating culture variables and production-enhancing strategies [24,27]. Optimization commonly involves modifications to culture medium composition, plant growth regulators, nutritional factors, environmental conditions, and culture duration, whereas elicitation has emerged as one of the most extensively investigated approaches for stimulating secondary metabolite accumulation across diverse in vitro plant systems [7,28,29]. Quantitative monitoring of metabolite accumulation throughout these optimization procedures enables objective evaluation of the effects of individual strategies, facilitating the identification of conditions that effectively enhance metabolite production. Other production-oriented approaches, including precursor feeding, selection of highly productive lines, two-stage cultivation systems, and metabolic engineering, further illustrate that optimization extends beyond culture maintenance and focuses on progressively improving the productive performance of established platforms [30,31].

Production reliability was considered supported when the study provided complementary experimental evidence indicating that the established in vitro culture system maintained quantifiable production of the target metabolite under defined, standardized, and controlled culture conditions. Supporting evidence included replicated quantitative measurements with statistical assessment, repeated production experiments, routine subculture accompanied by continued metabolite quantification, or operation under a standardized cultivation protocol or controlled production system with documented metabolite output [24,25]. Because the type and extent of evidence varied among the evaluated studies, these indicators were interpreted collectively rather than as interchangeable or individually mandatory criteria. Dedicated long-term stability assays, multi-cycle production studies, batch-to-batch assessments, and explicit demonstrations of process robustness during scale-up were considered indicators of advanced technological maturity rather than mandatory requirements for platform validation [28,32].

Table 1 summarizes these core attributes together with additional technological features associated with later stages of platform development, including scale-up studies, bioreactor validation, and advanced biotechnological approaches.

**Table 1:** Technological attributes identified from specialized reviews for assessing the degree of technological development of plant in vitro production platforms for secondary metabolite production.

Technological Assessment Category	Technological Attribute	Operational Definition	Representative Experimental Evidence	Representative Supporting Reviews
Core technological attributes supporting platform validation	Establishment of an in vitro production system	Successful establishment of an in vitro plant culture capable of producing the target secondary metabolite under controlled conditions.	Callus, cell suspension, adventitious roots, hairy roots, shoot cultures or organ cultures successfully established.	[4,6,11,24,33]
	Quantitative metabolite production	Quantitative determination of the target metabolite using validated analytical methods to objectively evaluate productive performance.	HPLC, UHPLC, LC-MS/MS, GC-MS, spectrophotometric assays; metabolite concentration (mg g^−1^ DW, mg L^−1^), productivity and yield.	[24,26,28,31]
	Optimization of culture conditions	Systematic evaluation of culture variables and production-enhancing strategies to improve metabolite concentration, productivity and yield.	Optimization of culture medium, plant growth regulators, nutritional factors, elicitation, precursor feeding, environmental conditions, culture duration, selection of highly productive lines.	[7,26,28,34]
	Production reliability	Ability of an established in vitro culture system to maintain quantifiable production of the target metabolite under defined, standardized, and controlled culture conditions, as supported by complementary experimental evidence	Replicated quantitative measurements with statistical assessment; repeated production experiments; routine subculture accompanied by continued metabolite quantification; or standardized cultivation protocols and controlled production systems with documented metabolite output.	[11,24,25,26,27,28,31]
Technological attributes reflecting advanced platform development	Metabolite yield relative to the donor plant	Comparison of metabolite accumulation between in vitro cultures and the donor plant to evaluate productive performance under controlled conditions.	Metabolite concentrations equal to or higher than those in donor plant tissues.	[2,6,35]
	Scale-up potential	Demonstration that metabolite production can be successfully transferred from laboratory cultures to larger-scale production systems.	Pilot-scale cultures; transition from flasks to bioreactors; large-volume production systems.	[25,30,36]
	Bioreactor validation	Demonstration of efficient metabolite production under controlled bioreactor conditions.	Stirred-tank, airlift, temporary immersion, nutrient sprinkle and wave bioreactors producing target metabolites.	[25]
	Advanced biotechnological approaches	Integration of molecular and systems biology tools to improve metabolite production and platform performance.	Metabolic engineering, CRISPR/Cas, multi-omics, metabolic flux analysis, transcription factor engineering.	[1,11,31]

The technological attributes presented in this table were identified through an analysis of review articles on plant in vitro production of secondary metabolites. The four core attributes supporting platform validation are further discussed in the text because, within the operational scope of this review, they represent the minimum complementary experimental evidence used to support platform validation. The additional attributes presented in Table 1 are representative rather than exhaustive and reflect more advanced stages of technological development frequently reported in the literature. Long-term production stability, multi-cycle or batch-to-batch performance, and process robustness during scale-up were likewise interpreted as indicators of advanced technological maturity rather than mandatory criteria for platform validation. Representative supporting reviews are provided as examples rather than as an exhaustive bibliography. Abbreviations: HPLC, High-Performance Liquid Chromatography; UHPLC, Ultra-High Performance Liquid Chromatography; LC-MS/MS, Liquid Chromatography–Tandem Mass Spectrometry; GC-MS, Gas Chromatography–Mass Spectrometry; DW, Dry Weight.

Based on the synthesis of technological attributes consistently reported across specialized reviews, the present review operationally defines a validated plant in vitro production platform as a culture system supported by complementary experimental evidence demonstrating (i) successful establishment of the in vitro culture system, (ii) quantitative determination of the target metabolite, (iii) optimization of culture conditions or production-enhancing strategies, and (iv) complementary experimental evidence of production reliability under defined, standardized, and controlled culture conditions. Together, these core attributes indicate that the system has progressed beyond the demonstration of biosynthetic potential and can be evaluated as a production-oriented platform for secondary metabolite generation. Additional attributes, including metabolite yield relative to the donor plant, scale-up potential, bioreactor validation, and advanced biotechnological approaches, are interpreted as indicators of advanced technological maturity rather than mandatory criteria for platform validation [11,24,26].

The distinction between core and advanced attributes was based on their analytical role in platform assessment. The four core attributes were considered the minimum complementary evidence needed to demonstrate biological feasibility, measurable metabolite production, experimental responsiveness to optimization, and maintenance of production performance under standardized culture conditions, whereas comparison with the donor plant, scale-up, bioreactor validation, and advanced biotechnological approaches were interpreted as indicators of subsequent technological development [11,24,25,26,30]. This operational framework is intended to support the comparative assessment of published experimental evidence rather than to serve as a universal regulatory or industrial validation standard; consequently, classifications depend on the completeness and quality of the information reported in the source studies, and the absence of published evidence was interpreted as insufficient evidence rather than proof that a platform lacks a particular capability. The scope of the framework is limited to the experimental and technological development of in vitro production systems and does not include economic feasibility, regulatory compliance, or commercial readiness.

Within this framework, platforms that do not meet all core validation attributes are classified as partially validated or emerging according to the level of experimental evidence available. A partially validated platform is defined as a culture system in which establishment and quantitative metabolite production have been demonstrated, but the available evidence remains insufficient to confirm systematic optimization and complementary experimental evidence of production reliability under defined, standardized, and controlled culture conditions. These systems provide measurable evidence of productive performance, but they still require further experimental development before being considered fully validated. By contrast, an emerging platform corresponds to a culture system in which the available experimental evidence remains limited to the successful establishment of the in vitro culture system, with or without preliminary metabolite characterization or initial quantitative assessment. Although these systems demonstrate potential for future metabolite production, further studies are required to establish their productive performance through quantitative evaluation, systematic optimization, and production reliability (Table 2).

**Table 2:** Technological assessment of plant in vitro production platforms for secondary metabolite production in tropical and subtropical plant species based on the validation framework proposed in Table 1.

Species (Family)	Native Distribution	In Vitro Platform	Target Metabolite	Analyte Concentration	Culture Optimization	Production Reliability	Technological Category	References
*Andrographis paniculata* (Acanthaceae)	Tropical	Leaf callus culture	Andrographolide	0.96207 ± 0.00027 mg g^−1^ DW by HPLC after 5 mg L^−1^ chitosan	Optimization of surface sterilization, plant growth regulator combinations, chitosan elicitation and elicitor concentration	Replicated HPLC quantification with statistical assessment under defined culture conditions; production stability across successive culture cycles was not assessed.	Partially validated	[37]
*Azadirachta indica* (Meliaceae)	Tropical	Hairy root culture	Azadirachtin	7.47 mg g^−1^ DW, 70.42 mg L^−1^ and 2.81 mg L^−1^ day^−1^ after cholesterol precursor feeding	Optimization of multiple elicitors (methyl jasmonate, salicylic acid, chitosan, yeast extract, fungal elicitors, metal salts, UV) and precursor feeding (cholesterol, squalene, IPP, GPP, acetate and MAL)	Selected transformed hairy root line with defined growth kinetics and standardized culture protocol; however, production stability across successive production cycles or scale-up was not experimentally demonstrated	Partially validated	[38]
*Bryophyllum* spp. (Crassulaceae)	Tropical	Cell suspension culture	Phenolic compounds	Eight phenolic subclasses semiquantified by UHPLC-QTOF/MS.	Optimization of SA, MeJA, genotype and elicitor combinations using neurofuzzy modeling	Triplicate elicitation experiments with statistically assessed UHPLC-QTOF/MS semiquantification; successive-subculture production stability was not evaluated	Partially validated	[39]
*Camellia sinensis* (Theaceae)	Tropical–Subtropical	Cell suspension culture, 5 L bioreactor	Catechins, polyphenols and caffeine	Total polyphenols 33.87%; total catechins 22.85%; caffeine 4.66% after 20 mM shikimic acid	Optimization of cell isolation, plant growth regulators, growth phase, precursor feeding and bioreactor culture	Cell suspension culture established under standardized conditions and successfully transferred to a 5 L bioreactor for controlled metabolite production	Validated	[40]
*Carica papaya* (Caricaceae)	Tropical	Callus culture	Phenolic compounds and flavonoids	TPC 44.24 mg GAE g^−1^ DW; TFC 23.11 mg rutin g^−1^ DW	Optimization of callus induction and extraction conditions	Callus established and analyzed; no optimized production, stability or repeated cycles	Emerging	[41]
*Catharanthus roseus* (Apocynaceae)	Tropical	Cell suspension culture	Vinblastine; Vincristine	Vinblastine ≈ 0.0024 mg g^−1^ DW; Vincristine ≈ 0.00125 μg g^−1^ DW by HPLC after combined *Trichoderma harzianum* + sodium nitroprusside elicitation (48 h)	Optimization of explant type, plant growth regulators, suspension establishment, elicitor combinations and harvest time	Growth kinetics and replicated HPLC quantification with statistical assessment under defined suspension-culture conditions; long-term stability was not assessed.	Validated	[42]
*Catharanthus roseus* (Apocynaceae)	Tropical	Callus culture	Catharanthine; Vinblastine; Vincristine	Catharanthine 0.00501 ± 0.00167 mg g^−1^ FW and vinblastine 0.00196 ± 0.00030 mg g^−1^ FW; vincristine 0.00471 ± 0.000005 mg g^−1^ FW	Optimization of explant source, PGR combinations, callus biomass, alkaloid accumulation and expression of TIA pathway genes	Short-term production quantified in optimized callus cultures with biological replicates and statistical analysis; production stability across successive subcultures or repeated production cycles was not directly demonstrated	Partially validated	[43]
*Celosia* *argentea* (Amaranthaceae)	Tropical	Cell suspension culture	Betacyanins, betaxanthins and total betalains	Total betalains up to 4.99 mg g^−1^ DW	Optimization of growth kinetics, harvest stage and elicitation	Two independent experiments with five replicates under defined culture conditions; successive-cycle production stability was not evaluated.	Partially validated	[44]
*Cullen corylifolium* (Fabaceae)	Tropical–Subtropical	Callus culture	Psoralen; Daidzein; Genistein	Psoralen 5.33783 ± 0.00633 mg g^−1^ FW; daidzein 1.715973 ± 0.00881 mg g^−1^ FW; genistein 2.43874 ± 0.00393 mg g^−1^ FW	Optimization of SA, chitosan and proline elicitation	Callus maintained in 30-day cycles; no stability between cycles	Partially validated	[45]
*Dipterocarpus alatus* (Dipterocarpaceae)	Tropical	Callus culture	Multiple bioactive metabolites	0.0011–0.9914 mM g^−1^ FW by H-NMR metabolomics	Optimization of TDZ, 2,4-D and BA for callus induction and metabolite profiling	Quantified after optimized callus induction; no stability, repeated cycles or scale-up	Partially validated	[46]
*Ehretia asperula* (Boraginaceae)	Tropical	Cell suspension culture	Rosmarinic acid	197.6 mg L^−1^	Optimization of aggregate size, glucose, two-stage culture and chitosan	No evidence of long-term production stability	Partially validated	[47]
*Embelia ribes* (Primulaceae)	Tropical–Subtropical	Organogenic and embryogenic callus cultures	Embelin	6.44% embryogenic callus; 5.72% organogenic callus by RP-HPLC after chitosan elicitation	Optimization of callus type, plant growth regulators, chitosan, salicylic acid and methyl jasmonate elicitation; regeneration and acclimatization	Organogenic and embryogenic callus cultures maintained through routine subculture with reproducible metabolite production under standardized culture conditions	Validated	[48]
*Fagonia indica* (Zygophyllaceae)	Tropical–Subtropical	Callus culture	Betulinic acid; Nahagenin; Caffeic acid	Betulinic acid 1573.71 ± 37.01 mg g^−1^ DW by HPLC after 250 mM NaCl treatment	Optimization of NaCl and Na_2_CO_3_ elicitation, biomass production, phenolics, flavonoids, antioxidant and anti-inflammatory activity	Short-term metabolite production demonstrated in optimized callus cultures; no evidence of production stability across successive subcultures, repeated production cycles or scale-up	Partially validated	[49]
*Gardenia jasminoides* (Rubiaceae)	Subtropical	Cell suspension culture	Geniposide; Genipin	Geniposide 3.9183 mg g^−1^; genipin 2.7796 mg g^−1^	Optimization of chitosan, MeJA, elicitor combinations and exposure time	No evidence of long-term production stability	Partially validated	[50]
*Gloriosa superba* (Colchicaceae)	Tropical–Subtropical	Hairy root cultures	Colchicine	0.0301 ± 0.00082 mg g^−1^ DW by HPLC	Optimization of microbial elicitors, elicitor concentration and exposure time	Stable transformed hairy root cultures maintained under standardized culture conditions; genetic transformation confirmed by rolB PCR	Validated	[51]
*Hybanthus enneaspermus* (Violaceae)	Tropical–Subtropical	Cell suspension culture	L-DOPA	16.82 mg g^−1^ DW by HPLC	Optimization of precursor feeding, polyamine elicitation, precursor concentration and exposure time	L-DOPA production assessed in three independent experiments under defined culture conditions; stability across successive sub-cultures was not evaluated.	Partially validated	[52]
*Impatiens balsamina* (Balsaminaceae)	Tropical	Callus culture	Total phenolics, total flavonoids, total tannins	TPC 6.59 ± 0.20 mg GAE g^−1^ DW; TFC 2.97 ± 0.04 mg QE g^−1^ DW; TTC 6.63 ± 0.07 mg TAE g^−1^ DW	Optimization of callus induction using 2,4-D	Callus established; no metabolite-production optimization or stability	Emerging	[53]
*Justicia gendarussa* (Acanthaceae)	Tropical	Callus culture	Phenolics, flavonoids and terpenoids	TPC 108.8 mg GAE g^−1^ DW; TFC 130.4 mg QE g^−1^ DW	Machine learning-assisted PGR optimization with experimental validation	Optimized callus production, but no long-term stability, repeated cycles or scale-up	Partially validated	[54]
*Mitragyna speciosa* (Rubiaceae)	Tropical	Cell suspension culture	Mitragynine	13.226 ± 1.98 mg L^−1^ by HPLC following 3 μM tryptophan feeding	Optimization of PGRs, sucrose, elicitation, precursor feeding and harvest time	Sequential subcultures, defined growth kinetics and consistent HPLC quantification	Validated	[55]
*Rubus fraxinifolius* (Rosaceae)	Tropical	Callus culture	Phenolic compounds; flavonoids	26.48 mg GAE g^−1^ DW TPC; 20.98 mg QE g^−1^ DW TFC	Optimization of yeast extract concentration, biomass accumulation, phenolic and flavonoid production, antioxidant activity and metabolite profiling	Replicated metabolite quantification with statistical assessment under defined culture conditions; production stability across successive culture cycles was not evaluated.	Partially validated	[56]
*Simmondsia chinensis* (Simmondsiaceae)	Subtropical	Callus culture	Phytochemical profile	Not quantitatively reported; relative GC-MS peak areas only	Optimization of callus induction through NAA and TDZ	Chemical characterization only; no quantitative production, stability or validation	Emerging	[57]
*Vitex negundo* (Lamiaceae)	Tropical–Subtropical	Cell suspension culture	Vitexin	8.97 mg g^−1^ DW by HPLC under 5% sucrose	Optimization of callus induction, NAA, MS strength, carbon source, sucrose, dark incubation and growth kinetics	Sequential subcultures, standardized suspension culture, defined growth kinetics and reproducible HPLC quantification following culture optimization	Validated	[58]
*Withania somnifera* (Solanaceae)	Tropical–Subtropical	Root cell suspension culture	Withanolide A	0.1220 ± 0.00052 mg g^−1^ culture filtrate; 0.01029 ± 0.00045 mg g^−1^ mycelial extract	Optimization of fungal elicitors and exposure time	Periodic subculture, but no direct long-term stability or scale-up	Partially validated	[59]
*Zanthoxylum armatum* (Rutaceae)	Subtropical	Shoot culture	Phenolic compounds	Chlorogenic acid 11.75 mg g^−1^ DW; gallic acid 9.96 mg g^−1^ DW; EGCG 10.45 mg g^−1^ DW; *p*-coumaric acid 8.63 mg g^−1^ DW	Optimization of sucrose, proline, SA, NaCl, pH and elicitors	No long-term stability or scale-up reported	Partially validated	[60]

Abbreviations: PGRs, Plant Growth Regulators; NAA, α-Naphthaleneacetic acid; MS, Murashige and Skoog; FW, Fresh Weight; DW, Dry Weight; HPLC, High-Performance Liquid Chromatography; TIA, Terpenoid Indole Alkaloids; L-DOPA, L-3,4-dihidroxifenilalanine; IPP, Isopentenyl pyrophosphate; GPP, Geranyl pyrophosphate; MAL, Malonate; GAE, Gallic Acid Equivalents; TPC, Total Phenolic Content; QE, Quercetin Equivalents; TFC, Total Flavonoid Content; SA, Salicylic Acid; MeJA, Methyl Jasmonate; EGCG, Epigallocatechin Gallate; H-NMR, Proton Nuclear Magnetic Resonance; TDZ, Thidiazuron; BA, Benzyladenine.

Application of the proposed validation framework to the evaluated studies showed that most in vitro production platforms accumulated experimental evidence corresponding to intermediate stages of technological development rather than fully validated production systems (Table 2). Most partially validated platforms incorporated quantitative metabolite determination and culture optimization following successful culture establishment, whereas explicit evidence of production reliability under standardized culture conditions was reported less consistently in this technological category. Both differentiated and undifferentiated culture systems were represented among the validated platforms, with cell suspension cultures constituting the predominant production system, whereas hairy root and organogenic callus cultures were also identified. In contrast, partially validated platforms encompassed a broader diversity of differentiated and undifferentiated culture systems, while all emerging platforms were based exclusively on callus cultures.

Culture optimization was consistently incorporated in both validated and partially validated platforms, irrespective of the in vitro culture system or target metabolites. The most frequently adjusted culture parameters included plant growth regulator combinations, elicitation treatments, precursor feeding, carbon source composition, harvest time and growth kinetics. Among the elicitation strategies, methyl jasmonate, salicylic acid and chitosan were the most frequently reported. More specialized approaches, including bioreactor cultivation, aggregate-size control, metabolomics-guided optimization, two-stage culture systems and machine learning-assisted optimization, were reported less frequently.

Although evidence of production reliability was identified across several partially validated platforms, the level of experimental support differed from that observed in validated production systems. Partially validated platforms frequently documented quantified metabolite production after culture establishment and optimization under defined in vitro conditions, whereas validated platforms more often incorporated evidence of long-term culture performance, successive production cycles, standardized production protocols, or controlled production systems [4,34]. The distinction was therefore associated less with metabolite detection or short-term optimization than with evidence that production persisted across standardized and repeated operation. This pattern distinguished partially validated platforms from validated platforms in the reviewed dataset.

Plant cell cultures are dynamic biological systems in which cellular populations continuously change throughout prolonged in vitro cultivation [61]. Rather than behaving as homogeneous production units, cultured cells progressively develop physiological, genetic, and metabolic heterogeneity through processes such as somaclonal variation, differential physiological adaptation, and cellular aggregation. These processes also generate, particularly in suspension cultures, distinct cellular microenvironments that influence nutrient availability, oxygen diffusion, cellular differentiation and metabolic activity, ultimately producing heterogeneous biosynthetic responses within the same culture [62]. In turn, sustaining consistent secondary metabolite production across successive culture cycles becomes substantially more demanding than demonstrating metabolite accumulation under a single set of experimental conditions, since long-term productivity depends on maintaining stable production performance despite the continuous biological evolution of the culture [28].

The predominance of validated cell suspension platforms observed in the evaluated studies may reflect both their biosynthetic competence and their suitability for systematic optimization. Cell suspension cultures provide relatively homogeneous, manipulable, and scalable cell populations, facilitating process standardization under controlled conditions. Their organization also allows medium composition, nutrient availability, plant growth regulators, elicitors, aeration, pH, temperature, and cultivation time to be adjusted systematically to regulate cell growth, physiological status, and secondary metabolism [25]. However, this advantage is not universal. Some specialized metabolites depend on tissue-specific pathways, subcellular compartmentalization, and coordinated metabolism among specialized cell types or organs. In *Catharanthus roseus*, for example, spatial separation of terpenoid indole alkaloid biosynthesis and transport limits the production of these compounds in undifferentiated cultures. Differentiated platforms such as hairy roots may therefore be more suitable when the target pathway depends on organ-specific organization while also offering genetic stability and compatibility with controlled cultivation and scale-up [12,63]. Platform selection should first account for the biological organization required by the target pathway and then for the capacity of the culture system to support reproducible operation, optimization, and scale up [33].

This intrinsic biological variability provides a mechanistic explanation for why production reliability represents one of the most demanding attributes during technological platform development. Demonstrating reliable production requires experimental evidence that metabolite production remains reproducible despite the dynamic behavior of plant cell populations, thereby reflecting biological stability rather than isolated production success. Consistent with recent reviews emphasizing reproducibility, standardized culture systems, and robust production processes as prerequisites for technological development, the predominance of partially validated platforms observed in the present review suggests that demonstrating stable and reproducible metabolite production remains substantially more challenging than culture establishment, metabolite quantification, or culture optimization considered individually [27,32].

Following successful culture establishment, plant in vitro production systems frequently exhibit relatively low metabolite productivity [64], making culture optimization an essential stage in technological platform development rather than an optional experimental refinement [65]. Among these optimization approaches, elicitation has become one of the most widely adopted strategies because elicitors mimic biotic or abiotic stress signals that activate cellular regulatory networks controlling specialized metabolic pathways [12]. Rather than acting directly on metabolite accumulation, they stimulate signaling and transcriptional processes that ultimately enhance secondary metabolite biosynthesis, predominantly through methyl jasmonate, salicylic acid and chitosan treatments [25]. Accordingly, culture optimization represents a progressive technological stage that bridges successful culture establishment with subsequent technological advancement of the production platform.

Despite the widespread implementation of optimization strategies, their development remains largely empirical because the regulatory networks governing secondary metabolite biosynthesis are not yet fully understood. Recent reviews emphasize that elicitor-induced responses involve complex signaling pathways and transcriptional reprogramming that vary among species, culture systems and target metabolites, limiting the rational design of universally applicable optimization protocols [12,66]. These limitations are particularly evident for biotic elicitors, whose inherent biological complexity, variable responses and practical constraints continue to hinder their standardization across production systems. Advances in transcriptomics, metabolomics and other omics-based approaches are expected to improve the understanding of these regulatory mechanisms, facilitating the transition from empirical optimization toward more rational and predictive strategies for platform development [67,68].

The present review distinguishes between species-level scientific knowledge and platform-level technological validation when assessing the development of plant in vitro production systems. While species-level scientific knowledge reflects the breadth of biological, biochemical and biotechnological research accumulated for a given species, platform-level technological validation depends on complementary experimental evidence demonstrating that an in vitro culture system satisfies the technological attributes summarized in Table 1. *Catharanthus roseus* illustrates this distinction particularly well. As one of the best-characterized medicinal plant species from tropical regions, it has served as a reference system for studies on terpenoid indole alkaloid biosynthesis, metabolic regulation, genetic transformation, metabolic engineering and plant tissue culture [69]. Nevertheless, researchers continue to identify important challenges associated with the complexity of biosynthetic regulation, the instability of high-producing cell lines during prolonged cultivation, and the persistence of biological and technological constraints affecting the long-term stability of metabolite production [63,70]. Consistent with this distinction, the evaluated experimental evidence supports different levels of technological development among *C. roseus* platforms: the cell suspension system was classified as validated, whereas the callus-based system remained partially validated [42,43]. This distinction highlights the value of evaluating plant in vitro production platforms using standardized technological criteria instead of relying on the volume or diversity of published research, thereby providing a more consistent framework for assessing technological development across tropical and subtropical species.

Future progress in plant in vitro production platforms will require a shift from predominantly empirical optimization toward knowledge-driven development based on deeper mechanistic understanding of secondary metabolite biosynthesis. Transcriptomics, metabolomics, metabolic engineering, and other systems biology approaches can identify regulatory networks governing metabolite accumulation and guide more rational and predictable culture optimization. Combined with transcription factor engineering and precise genome editing, these approaches may also redirect metabolic flux according to the biological requirements of the target metabolite [36,70]. However, mechanistic knowledge alone will not produce technologically mature platforms; it must be integrated with complementary engineering strategies throughout platform development [25]. Future advances should therefore be evaluated not only by increases in metabolite yield but also by the evidence they provide of reliable platform performance [27,32,71].

Achieving higher levels of technological maturity will ultimately require complementary advances in process engineering to translate biologically productive cultures into reliable and scalable production platforms. Long-term validation of production performance, standardized cultivation protocols, bioreactor-based production systems, and advanced process monitoring and control strategies should be considered integral components of platform development rather than independent technological improvements. Additional process-engineering approaches, including two-stage cultivation strategies, cell immobilization systems, and other advanced cultivation configurations, may further enhance production stability by separating biomass accumulation from metabolite synthesis, facilitating continuous or repeated production cycles, and improving process scalability [71]. Rather than representing isolated technological innovations, these advances should collectively strengthen platform robustness, reproducibility and scalability, thereby supporting progression through successive stages of technological maturity. Applying standardized validation criteria to evaluate these advances will facilitate objective comparison among production systems, identify critical technological bottlenecks, and guide the rational development of increasingly mature production platforms [9,72].

## 4. Conclusions

The present review demonstrates that technological maturity of plant in vitro production systems should be understood as a progressive process resulting from the accumulation of complementary experimental evidence rather than from isolated experimental achievements. Although platform development encompasses biological, biotechnological and engineering dimensions, not all forms of experimental evidence contribute equally to technological progression. While some provide the evidence required to recognize an in vitro culture system as a validated production platform, others characterize more advanced stages associated with process intensification, industrial implementation and long-term operational performance. This progressive perspective provides a more objective basis for assessing technological maturity while distinguishing the scientific knowledge accumulated for a species from the developmental status achieved by its in vitro production system.

Application of this technological perspective revealed that most tropical and subtropical medicinal species have progressed beyond successful culture establishment, metabolite identification and initial optimization, yet relatively few have accumulated sufficient complementary evidence to support fully validated production platforms. These findings indicate that the principal challenge is no longer demonstrating the biosynthetic potential of plant in vitro cultures but developing reproducible production systems capable of maintaining stable metabolite production under standardized cultivation conditions. Consequently, future advances in this field will depend less on expanding descriptive phytochemical knowledge than on strengthening the technological maturity of existing production systems through greater process robustness, long-term production stability and standardized production practices.

## Data Availability

No new data were created or analyzed in this study. Data sharing is not applicable.
